# Book Review

**Published:** 1898-06

**Authors:** 


**Physiology. Students' Note-Book (for the Laboratory).** Part I. —*Physiological Chemistry*. By ARTHUR J. HALL, M.B. Pp. 47. London: Baillière, Tindall, and Cox. 1897.—Dr. Hall has arranged his work on a novel plan—the alternate pages are left blank for a student's notes on the reactions described on the opposite page. It must be confessed that some laboratory guides tend to assume the bulk and complexity of text-books. Such a plan as is here recommended has much in its favour; it is simple, and yet gives the pupil sufficient theory to make his practical work interesting.

**The Student's Guide to Medical Diagnosis.** By SAMUEL FENWICK, M.D., and W. SOLTAU FENWICK, M.D. Eighth Edition. Pp. xxxii., 468. London: J. & A. Churchill. 1897.—The eighth edition of a book needs little comment; it is obvious that the book is a popular one, and further that, since the first edition was written in 1869, it was necessary that the edition of 1897 should have been practically re-written. We are told that "almost all the old microscopical illustrations have been discarded for others of a more modern type"; but we think that the sphygmograms (page 32) might well be replaced by something better. An attempt to compress the outlines of pathology and the symptoms and physical signs of all the ordinary diseases into the compass of this volume is of necessity bound to do scant justice to some of them.

**Notes on Malaria in Connection with Meteorological Conditions at Sierra Leone.** By Surgeon-Major E. M. WILSON. Pp. 16. London: H. K. Lewis. 1897.—The results of the author's observations show that the East India regiments suffer considerably during the first year of service on the coast of Sierra Leone, and less in succeeding ones; that whites are more susceptible than coloured troops; that the greatest number of cases occur in the rainy season, the least in the dry; and that the most important factor is the relative humidity of the atmosphere. The author speaks from experience, and his suggestion that the coloured troops should be put on a ration allowance should be seriously considered by the authorities.

**The Pocket Therapist.** By THOS. STRETCH DOWSE, M.D. Pp. 192. Bristol: John Wright & Co. 1897.—The author makes so ample an apology for "this small and apparently trivial work," and for "its insufficiency and inadequacy," that we have little need to point out its errors of omission. It contains a record of much ripe experience, and therefore cannot fail to be useful to the inexperienced; but why the latter should not go to a complete text-book or a monograph for information we fail to see. There appears to be little need for a book like this, which in so small a space professes to be a guide to therapeutics, to health-resorts, poisons and their antidotes, and clinical diagnosis. We wonder what mysteries are hidden behind such names as Branalcane, Salycilic (*sic*) Valsol, Neboline.

**Diet Chart and Alimentary Index.** Suggested by M. J. KENNY. Bristol: John Wright & Co. 1897.—Each page of this book is a list of ordinary foods and drinks, with a space for marking those which are suitable to the case: the page is torn out and given to the patient. The list is by no means complete, and we fail to see on what principle the selection has been made. The alimentary index is inside the covers, and we venture to think that this is the most useful part of the book. Some formulæ for making broths, &c., are on the back of each chart. Surely the beef tea need not have the meat excluded by straining: why should not the whole of the meat be retained?

**Gynæcology in Berlin.** By H. MACNAUGHTON-JONES, M.D. Pp. 28. London: John Bale, Sons and Danielsson, Limited. 1897.—Reprinted from the *British Gynæcological Journal*, this forms an interesting little book, containing in a concise form many methods which are not usually practised by English surgeons. The observations are taken from the clinics of Martin, Olshausen, and Landau, whose work is in many respects original. It is well worth reading, and is freely illustrated with helpful plates.

**Our Baby.** By Mrs. LANGTON HEWER. Fifth Edition. Pp. vii., 140. Bristol: John Wright & Co. 1897.—We so recently reviewed the previous edition, that a notice need hardly be given to this work. The book is "revised," but is practically the same as the last edition. It contains much useful information; but, like most publications of this sort—especially later editions—too much amateur physicking is recommended.

**A Handbook of the Diseases of the Eye.** By HENRY R. SWANZY, M.B. Sixth Edition. Pp. xviii., 608. London: H. K. Lewis. 1897.—The author has thoroughly revised his book, and has not materially increased its bulk. This is accounted for, to some extent, by the fact that the pages on "Some Elementary Optics," found in the first chapter of previous editions, have been omitted. The practitioner will especially appreciate Chapter xvii., in which the ocular diseases and symptoms liable to accompany diffuse organic brain lesions and diseases of the spinal cord have been treated more fully, whilst there has also been introduced an account of the "Functional Derangements of Vision." An excellent account of tumours of the orbit and neighbouring cavities is to be found in Chapter xix., the purely pathological aspect of that class of diseases being avoided. The aim of the author to say all he ought, without saying all he might, has been successful; the result being an exceedingly well-written book, that, taking into consideration its size, cannot, in our opinion, be excelled.

**A Handbook of Diseases of the Nose and Pharynx.** By James B. Ball, M.D. Third Edition. Pp. xii., 404. London: Baillière, Tindall and Cox. 1897.—We notice very few changes in the present edition of this work, which we had opportunity to notice favourably on a former occasion. The author has wisely refrained from adding materially to the length of the book, and has kept it within a handy compass. It now gives in concise form a most readable and trustworthy description of all the ordinary affections of the nose, naso-pharynx, and pharynx. We can recommend it as well suited for students or practitioners.

**Excretory Irritation, and the Action of Certain Internal Remedies on the Skin.** By David Walsh, M.D. London: Baillière, Tindall and Cox. 1897.—This little work is made up of three elements,—a graduation thesis of 43 pages, a paper of 11 pages read at the Third International Congress of Dermatology, and a publisher's list of 48 pages. The two papers are on a similar topic, one which Dr. Walsh has made his own, the association between excretion and dermatitis. That the skin suffers as one of a group of excretory organs is abundantly demonstrated, and the action of certain internal remedies on the skin is explained. It is interesting to note "that any internal irritant which inflames the skin may be suspected of inflaming other excretory outlets," and "that the law of excretory irritation has a wide application to general pathology."

**The Treatment of Lupus.** By Balmanno Squire, M.B. Pp. 62. London: J. & A. Churchill. 1897.—The principal object of this booklet seems to be the recommendation of "multiple linear scarification" by means of a mincing machine with sixteen blades, set $\frac{1}{32}$ inch apart. From our own experience—and from enquiries—no one seems to have come across any of the "very many cases of lupus" so completely treated "that the patients have remained absolutely free from the disease for a series of years." Dr. Squire appears to overlook the fact that many cases of so-called lupus erythematosus are but the incipient stages of lupus vulgaris.

**A Plea for a State Medical Service.** By James Erskine, M.B. Pp. 20. Glasgow: John Morgan & Co. 1897.—Dr. Erskine's plea contains many pleas, and space will not permit us to criticise them. Though many of the opinions expressed by the author are fully warranted by facts, we do not think the remedies he recommends would cure the numerous evils that exist. We are not educated sufficiently yet in socialism—to use the term in its widest sense—to adopt a State medical service.

**Thirty-sixth Annual Report of the Cincinnati Hospital.** Cincinnati: *The Commercial Gazette* Job Print. 1897.—The most noteworthy topic we have noticed in this volume of 276 pages is a report on a series of cases of syphilis treated with Coley's mixed toxins. In some of the cases the result was good, the drug acting as quickly and as well as mercury. The report shews "the present of the remedy—its past is very young, its future yet unveiled. Time alone can fathom the outcome of these cases."

**King's College Hospital Reports.** Vol. III. London: Adlard and Son. 1897.—Old King's men will take more special interest in this volume in consequence of the obituary notice of Sir George Johnson, and the description of the new ophthalmological theatre which forms his memorial. Dr. Raymond Crawfurd, in a good paper on Graves's disease, emphasises the neurotic theory as opposed to the popular one of thyroid toxæmia, and he has great expectations that the influence of suggestion on the disordered mental state may prove to be of value in the treatment of the disease. The record of "what old King's men are doing" shows that they are well maintaining the credit of their school.

**Saint Bartholomew's Hospital Reports.** Vol. XXXII. London: Smith, Elder, & Co. 1897.—*St. Bartholomew's Reports* is, as usual, full of interest. The first two papers are reminiscences of two well-known surgeons: one, Professor Humphry, of Cambridge, had been a student at the hospital in the days when Sir James Paget was curator of the museum; the other, Mr. Morrant Baker, a man well known to all students of more modern times. These papers will be read with a sad interest by all old Bartholomew's men. One of the most striking and almost unique papers in this volume is a collection of aphorisms of Dr. Gee. They have been collected and edited by Mr. Horder and revised by their author. When reading them many will feel that they are again standing around the bedside of the patient and listening to Dr. Gee's remarks on the diagnosis and treatment of the case. Some cases of enteritis which simulated intestinal obstruction are recorded by Dr. Mitchell. In three out of the four cases the patients were operated on for intestinal obstruction. A very valuable paper on hernia of the vermiform appendix has been contributed by Mr. McAdam Eccles, in which he has given the records of a large number of such cases. As most of the papers are well worth reading, it seems unnecessary to refer to them in detail. They are on both medical and surgical topics. The volume contains the usual table of cases, with interesting remarks on the more important ones, and a description of specimens added to the museum.

**The Westminster Hospital Reports.** Vol. X. London: J. & A. Churchill. 1897.—This volume is a welcome addition to the series, for it contains the records of an unusually interesting number of cases of the varied description necessarily found in "Reports." The accounts of *post-mortem* examinations are very full; but we think it desirable that in future issues a greater prominence should be given to the clinical aspects of the disease by more fully tabulating some of the chief symptoms found in the various diseases—as in Table F, which treats of lobar pneumonia.

**Transactions of the College of Physicians of Philadelphia.** Third Series. Vol. XVIII. Philadelphia: Printed for the College. 1896.—The discussions and papers collected together in these *Transactions* make a very readable volume, and are of very considerable interest. Brain-surgery is, perhaps, the most important subject dealt with, and is liberally represented. Dr. W. W. Keen speaks hopefully of the surgical future of intra-cranial tumours, and makes some interesting observations on the attempts to secure bony occlusion of trephine-openings; Dr. Mills has a thoughtful paper on diagnosis; and, besides other formal papers contributed to the regular discussion, there are reports of isolated cases. A discussion on the Röntgen rays is included, but this is hardly as satisfactory; the ground is too shifting, and some of the conclusions are already out of date. Two cases of the rare condition of floating liver are reported; and there is a good sprinkling of interesting clinical material. The printer's work has been excellently done.

**Transactions of the New York Academy of Medicine.** Second Series. Vol. XI. Printed for the Academy. 1895.—This is a stout volume of over 600 pages, which contains many papers on all branches of medicine read before the Academy in 1894. A reflection which strikes us, on looking through these *Transactions* two years subsequently, is what a small amount of knowledge is added to our stock by all this work. Thus, there are three elaborate papers on scurvy in infants, not one of which goes beyond what Cheadle and Barlow had already established. The papers were no doubt interesting at the time, insomuch as they served to impress on the audience the clinical features and treatment of the malady, but seem hardly worth printing. There is the usual sprinkling of what can only be called oddities of medical thought. Thus, Dr. Achilles Rose advocates Greek as the international language of physicians. Again, Dr. Van Fleet examined, by a somewhat primitive method, the eyes of a number of children in reformatories, and finding a great number of errors in refraction, comes to the conclusion that defective eyesight is a factor in the production of crime. He does this, too, without attempting to determine whether the percentage of children with defective vision in these reformatories is greater than that in public schools and institutions not reformatories. Such are the whimsicalities of specialism.

**Transactions of the Medical Society of the State of New York.** Published by the Society. 1897.—This report contains no less than forty-six communications on various subjects, some of which are of considerable interest both medically and surgically. A large space is devoted to papers on the relation of impure water to disease, typhoid fever taking the greater share of attention. Thus there are papers on the clinical diagnosis of the disease, the life-history of the germ, the disease in infants, the treatment of the disease, and methods of disinfection of typhoid excreta. Surgically there is a complete exposition of hereditary syphilis by Dr. A. Jacobi, and a paper by Dr. A. M. Phelps on the management of club-foot, which gives his later experiences with the operation which he devised and was the first to use. There are papers on the modern treatment and prognosis of heart disease, on cough, on the advantages of strophanthus, on certain affections of the eye, on gauze drainage, and various conditions which require abdominal section, besides numerous other articles. The volume may be studied with profit by those engaged in any branch of the profession.

**Transactions of the Grant College Medical Society, Bombay.** Bombay: Printed at the *Tatva-Vivechaka* Press. 1897.—In spite of plague, famine, and earthquakes, the medical work of our Indian *confrères* goes steadily on, and this record, although carelessly sent out with plenty of misprints, and without title-page, table of contents, or index, gives much interesting information on the Bubonic Plague in Bombay. A mortality during the first four weeks of the outbreak ranging from 95 to 99 per cent. is in accord with what we know of the pathology of the disease, for which no efficient antidote appears yet to have come into use.

**Transactions of the American Gynecological Society.** Vol. XXI. Philadelphia: Wm. J. Dornan. 1896.—The science and art of gynæcology owes much of its advance to trans-Atlantic operators, and every volume published by the American Gynecological Society bears witness to the originality of its members. The present volume is in nowise behind its predecessors, and everyone interested in the subject will find in these pages a collaboration of the most careful and highly successful work in this branch of surgery. We cannot specify the different articles treating of so many varied subjects, but as a whole the book is characterised by profound research and literary merit, and the writers must be regarded as pioneers of the operative treatment of diseases of women.

**Transactions of the Obstetrical Society of London.** Vol. XXXVIII. London: Longmans, Green, and Co. 1897.—Of the many interesting communications contained in this volume, it is only possible to mention a few of the cases and papers contributed, one of the most important subjects dealt with being deciduoma malignum. Mr. Rutherford Morison and Dr. Herbert R. Spencer each reported a case. Mr. John D. Malcolm narrated a case of malignant disease of the uterus, with numerous deposits in the lungs, and death following an abortion; while Dr. T. W. Eden presented a paper, in which he criticised the whole subject, and pointed out several weak points in the cases of this affection described by continental and other observers, pointing out that plasmodia resembling the multinucleated syncytial masses have been found in sarcomata unconnected with pregnancy, and that the transition from placental villi to the structures composing these tumours has not been demonstrated; he states that proof of two points is necessary to the acceptance of the theory that these tumours are caused by the growth of decidual elements of partly maternal and partly fœtal origin, the first being the connection of the growth with a preceding pregnancy, and the second its development from structures which may be definitely recognised as placental relics. The interesting discussion which followed must be read in the original; but while the pathological changes which occur are debatable, the clinical manifestations are unmistakable, and point to the occurrence of a peculiarly rapid and fatal form of malignant disease apparently occurring in connection with pregnancy. Space does not permit a description of the other papers. The volume is well indexed, and contains some capital illustrations of naked-eye and microscopical changes occurring in the subjects under review.

**The American Year-Book of Medicine and Surgery.** London: The Rebman Publishing Co. (Ltd.). Philadelphia: W. B. Saunders. 1898.—The third number of this blue-cover year-book, being a digest of scientific progress and authoritative opinion in all branches of medicine and surgery, collected and arranged by numerous coadjutors, under the general editorial charge of Dr. George M. Gould, forms a volume of 1077 pages, nearly 200 less than last year, and we are informed that "with the growing clearness of conception, on the part of the editors, of the exact nature of the professional need there has been a corresponding recognition of the necessity of keeping the epitomization within the limits allotted to previous issues." It forms still a very large but handsome and well-illustrated volume, of which the publishers and editors may well be proud. The first subject dealt with is typhoid fever, and some twenty-seven pages are devoted to this topic. Last year we took occasion to comment upon the fact that the Widal test had been ignored, but now we come upon it as the most important contribution to the progress of internal medicine, and Drs. Pepper and Stengel state that "for our own part, we have become so thoroughly convinced of the value of the test that we should hesitate to diagnose typhoid fever in any case in which repeated examinations, made in approved fashion, failed to reveal agglutination." A table is given comprising over two thousand cases by various observers, and it demonstrates very conclusively the great diagnostic value of the reaction, correct results having been obtained in 96.5 per cent. of the cases. The diazo-reaction of Ehrlich has at last been considered worthy of note, and is reported to have "a negative diagnostic value," as also to be of some aid in prognosis, for, "if found in the 1st, 2d, or 3d days, or if it disappears by the end of the 2d or 3d week, the prognosis is favorable," whereas, "if constant to the end of the 4th week, it is more uncertain and relapse likely." In the domain of treatment the authors note the further advances of serum therapeutics; but although some favourable cases of treatment of typhoid by an antitoxic serum have been recorded, yet there is no certainty of success in this direction. The purgative method appears to be diminishing in popularity, but the cold-water treatment maintains its position, and operative interference in the 1 or 2 per cent. of cases of perforation has been successful in a few cases. In the case of tuberculosis there is as yet little certainty, notwithstanding the favourable reports of the followers of Maragliano, of Paquin, and of Koch. The best results seem to be always obtained by the persons who have produced the serum, but the results of experimental work in serum-therapy are more definite and satisfactory than the reports of its clinical use seem to indicate. It is said that "the Schott method for the treatment of heart-disease is still exciting considerable discussion. In general, the evidence is favorable, although warnings against its indiscriminate use are not lacking." The Röntgen rays have been employed for the determination of the alterations produced in the size of the heart, and Schott points out the necessity for a consideration of relative distances between the Crookes tube, the patient, and the photographic plate. The distance between tube and plate has always been one-half metre, experience having shown that this distance reproduces the natural size of the heart. Photographs and diagrams of the fluoroscopic image are not only a valuable addition to physical diagnosis, but they at the same time show in a most trustworthy way the process which takes place in the heart. The screen appears to give the best results.

**The Medical Annual.** Bristol: J. Wright & Co. 1898.—An enormous amount of information is contained in this volume. In fact it gives us all that is worth knowing about the medical and surgical novelties of the past year. The staff of contributors includes many men of great experience in their special departments of work, and they have well performed their heavy task of abstracting and editing. The volume is well illustrated, and is, in every way, a valuable book.

**Index-Catalogue of the Library of the Surgeon-General's Office, United States Army.** Second Series. Vol. I., A—Azzurri. Vol. II., B—Bywater. Washington: Government Printing Office. 1896-7.—These portions of this stupendous work include 22,078 author-titles, representing 12,510 volumes and 21,129 pamphlets. They also contain 13,658 subject-titles of separate books and pamphlets and 52,109 titles of articles in periodicals. Every page bears testimony to the arduous and well-executed task of the compilers, who are to be much congratulated on their valuable work. No library can do without this time-saving book of reference.

**The Philadelphia Medical Journal.** Philadelphia: The Philadelphia Medical Publishing Company.—So far from Dr. Gould's journalistic energy being used up by the production of his *Year-Book*, it seems on the contrary to have received fresh stimulus, for he has become the editor of the newly-founded *Philadelphia Medical Journal*, a weekly periodical which, in the opinion of its promoters, has "better professional aims and methods than have generally been customary," and which is going to decline any advertisements of secret preparations. The journal is decidedly one to take, for Dr. Gould impresses upon it the marks of his excellent individuality. We specially like the way in which "the latest literature" is presented; but it would be still more attractive if it were introduced by a classified index to the given abstracts. There does not seem sufficient reason for, or advantage in, the topsy-turvydom which puts the original articles at the end of the journal, as if they were the least important part of its contents.

**Smithsonian Report.** Washington: Government Printing Office. 1896.—The most interesting portion of this volume is the series of essays on a variety of scientific subjects which constitutes the general appendix. Amongst these are several sent in competition for the Hodgkins prizes, the most valuable of which was awarded to Lord Rayleigh and Professor Ramsay for their discovery of argon. Many of the papers here printed will be of special interest to readers of this *Journal*. A perusal of the secretary's report will show that this institution is doing most valuable work, both by assisting original research and by rendering easily accessible information as to recent progress in almost all branches of science. If the reports could be issued more promptly their value would be greatly enhanced.

**The Isle of Man Official Guide.** Pp. 80. Douglas: Brown & Sons, Limited. [N.D.]—Issued under the auspices and authority of the official board of advertising for the Isle of Man, this booklet gives all details such as are likely to be useful to intending visitors to the island, and cannot fail to be of service to such.

